# Fatigue as an Extra-Intestinal Manifestation of Celiac Disease: A Systematic Review

**DOI:** 10.3390/nu10111652

**Published:** 2018-11-03

**Authors:** Lars-Petter Jelsness-Jørgensen, Tomm Bernklev, Knut E. A. Lundin

**Affiliations:** 1 Department of Health Science, Østfold University College, N-1757 Halden, Norway; lars.p.jelsness-jorgensen@hiof.no; 2 Department of Gastroenterology, Østfold Hospital Trust Kalnes, N-1714 Grålum, Norway; 3 Department of Research and Innovation, Vestfold Hospital Trust, N-3103 Tønsberg, Norway; tomm.bernklev@medisin.uio.no; 4 Faculty of Medicine, Institute of Clinical Medicine, University of Oslo, N-0318 Oslo, Norway; 5 K.G. Jebsen Coeliac Disease Research Centre, University of Oslo, N-0318 Oslo, Norway; 6 Department of gastroenterology, Oslo University Hospital Rikshospitalet, N-0372 Oslo, Norway

**Keywords:** fatigue, energy, celiac disease, extra-intestinal manifestations

## Abstract

Celiac disease may present with a range of different symptoms, including abdominal problems in a broader sense, iron deficiency and “constant tiredness”. All of these symptoms should consequently lead the clinicians to consider celiac disease as a potential etiopathogenetic cause. Although the pathophysiology of celiac disease is well documented, the actual mechanisms for disease presentation(s) are less well understood. We here address the topic of fatigue in celiac disease. A systematic literature search identified 298 papers of which five met the criteria for full evaluation. None of the reviewed papers were of high quality and had several methodological weaknesses. We conclude that there is an unmet need to study the contributing factors and management of fatigue in celiac disease.

## 1. Introduction

Celiac disease is by definition an inflammatory disorder in the small intestine that is driven by dietary gluten from wheat, rye and barley [1]. The disease is often also termed an autoimmune disease due to a hallmark of the disease; autoantibodies to the endogenous enzyme tissue transglutaminase TG2. The crucial importance of intestinal inflammation and the most frequent presentation of the disease as a severe malabsorption syndrome led most clinicians to think of the disease as mainly an intestinal disorder. However, we now know that a very large proportion of the patients do not primarily display intestinal complaints, but that their disease presentation is more coloured by extra-intestinal manifestations [2,3]. In a recent review by Leffler et al. [3] this is well described, including anaemia, musculoskeletal, skin, neurological and organ-specific manifestations. Some of these manifestations are caused by the intestinal disorder, others may be caused by systemic inflammation and/or genetic overlap to other immune disorders [4]. Today we consider that symptoms like abdominal problems in the wider sense, unexplained iron deficiency and “constant tiredness” should all prompt the clinicians to consider celiac disease [5,6].

From our own clinical practice, we experience many patients with untreated celiac disease that suffer from fatigue. In most cases fatigue improves with diet, but far from always. In addition, these patients also present with a significant reduction in their health-related quality of life [2]. Fatigue is described as a “persistent, overwhelming sense of tiredness, weakness or exhaustion resulting in a decreased capacity for physical and/or mental work”. While fatigue may be a natural and transient part of life, a typical feature in chronic disease is that these symptoms are unrelieved by adequate sleep or rest [7]. The aetiopathogenesis of fatigue in chronic disease appears to result from a complex inter-relationship of biological, psychosocial and behavioural processes [8].

The aim of this review was consequently to address the aspect of fatigue in celiac disease and to systematically summarise the existing literature on this topic. 

## 2. Materials and Methods 

Multiple searches were undertaken using MEDLINE, CINAHL, EMBASE, Psychinfo, Academic Search Premiere, and Cochrane. Both medical subject heading (MeSH) searching and free-text searching were used to maximize citation retrieval (Table 1). The searches were performed independently by a university librarian, and one of the co-authors (L.-P.J.-J.). 

Due to the limited number of publications on fatigue in coeliac disease, no time limit was set for the papers. The searches were performed between April and June 2018, and the most recent search was performed on August 5th, 2018. The searches were limited to “humans”, “adult” and English language since there was no scope for translation. 

Studies with all types of designs were included if they had measured and reported data on fatigue in patients with celiac disease. Reviews, commentaries, abstracts/posters, case reports, protocols and letters to editors were excluded. The review was conducted in line with the PRISMA guidelines [9].

## 3. Results

**Figure 1:**
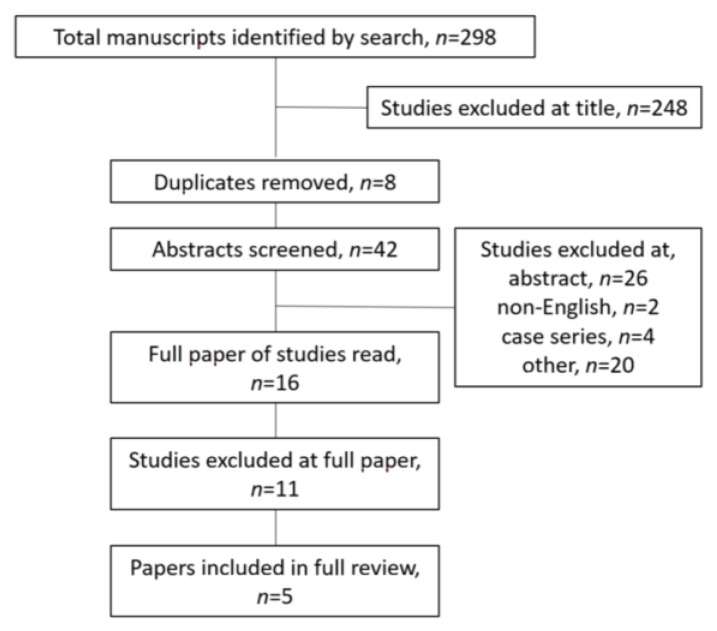
Citation retrieval and handling process.

The search yielded 298 references in total, of which 248 were excluded on title (Figure 1). After removing duplicates and screening the remaining 42 papers, a total of 16 papers were examined in full. Of these 16 papers, 11 did not report any specific methodology for fatigue measurement and were consequently excluded. Hence, a total of five papers were included in full review. Figure 1 describes the citation retrieval and the handling process in detail.

### 3.1. Quality Assessment

The quality of the included papers was assessed using the Joanna Briggs Institute Critical Appraisal Checklist, specific to the methodological design of each paper. The studies were classified as being of high, medium or low quality. A quality score was reduced if the paper did not define fatigue, report the sample size, if the sample size was judged inadequate according to study design, if the response rate was low or not reported, if the questionnaires used were not validated, if the methods and statistical analysis was insufficiently described or if there were indications of selective reporting. Two researchers (T.B./L.-P.J.-J.) performed quality assessment independently to ensure optimal assessment of the included papers. Based on these criteria, all of the reviewed papers had several methodological weaknesses, and none were judged to be of high quality. However, due to the low number of publications on fatigue and celiac disease, none of the papers were excluded from full-review based on quality.

### 3.2. Outline of the Included Papers 

In total, three papers assessed fatigue as the primary endpoint, one study investigated extra-intestinal symptoms and health-related quality of life (HRQoL), while one study assessed quality of sleep. An overview of the included papers is presented in Table 2.

### 3.3. Definition and Measurement of Fatigue

In two out of five papers, a definition or a more detailed description of fatigue was provided. Both Siniscalchi et al. [11] and Ciacci et al. [13] defined fatigue as ”difficulty in initiating or sustaining regular activities”. However, in none of these studies a reference to the definition were provided. 

In total, six different ways of measuring fatigue have been used in the five studies reviewed. Five of these instruments have only been used once. In addition, one study has used the sub-scale vitality from the health-related quality of life questionnaire SF-36. 

In a majority of the papers, there is lack of information about the validity and reliability of the instruments used to measure fatigue. While Häuser et al. [10] and Jordá et al. [12] provide references to adequate psychometrical testing of the SF-36, Giessener Symptom Checklist (GBB-24) and Daily Fatigue Impact Scale (D-FIS), none of the Italian studies provide clear reference to adequate testing. In fact, in two of the latter studies one of the instruments seem to have been labeled differently while referring to the same reference by Wessely et al. [15]. While Siniscalchi et al. [11] use the label Chronic Fatigue Syndrome (CFS) questionnaire, Ciacci et al. [13] use the label Verbal Scale for Asthenia. Moreover, when investigating the original study by Wessely et al. [15] in depth, it seems like this instrument was developed for this particular study and that the necessary tests for validity and reliability were not performed, and at least not published.

### 3.4. Prevalence of Fatigue and Its Associations

None of the reviewed studies present prevalence data, even though one of the aims in the study by Siniscalchi et al. [11] were to evaluate the prevalence of fatigue in celiac disease. When looking at fatigue in patients on normal versus gluten-free diet, results also differ. While Zingone et al. [14] and Siniscalchi et al. [11] found no significant differences, Jordá et al. [12] found that untreated patients reported significantly worse fatigue. In addition, when comparing celiac disease patients and healthy controls, both Häuser et al. [10] and Zingone et al. [14] found impaired scores in celiac disease. 

While Zingone et al. [14] found impaired sleep to be associated with increased fatigue, Jordá et al. [12] found that increased fatigue was associated with impaired HRQoL. Merely two studies [11,12] investigated potential socio-demographic and clinical factors associated with fatigue, finding that there is no association between fatigue and factors such as gender, age, or GI symptoms in celiac disease. While Jordá et al. [12] reported that lower haemoglobin levels were correlated with worse scores of the D-FIS fatigue scale, Siniscalchi et al. [11] were not able to identify any association.

### 3.5. Interventions to Alleviate Fatigue

Only one of the studies were designed as an intervention. The study by Ciacci et al. [13] aimed to investigate the effect on fatigue of a long l-Carnitine treatment in adult celiac disease patients. While there were no reports of serious adverse events, abdominal and skin problems were registered in a total of six patients (10%). Moreover, a large number of patients did not complete the study (*n* = 13), of which three were dropouts. The main finding is that fatigue scores were significantly more improved in the intervention versus placebo group. However, even though fatigue scores in the intervention group displayed a larger decrease than in the placebo group, patients in the intervention group had a higher fatigue scores than the placebo group at baseline (T0). Moreover, the mean fatigue VAS at the end of study (T2) was 2.40 (SD 1.80) versus 2.93 (SD 1.85) in the intervention versus placebo group, respectively. While Ciacci et al. does not report any measures of effect size, calculation of Cohens d [16] on the differences between the intervention and placebo group at T2 reveal a small effect size (0.29).

## 4. Discussion

In this review we were only able to identify 16 papers that, to some extent, had investigated fatigue in celiac disease. Of these, 11 papers [6,17,18,19,20,21,22,23,24,25,26] did not report any specific methodology concerning fatigue assessment. Of the remaining five papers [10,11,12,13,14], in which fatigue assessment had been described methodologically, merely three investigated fatigue as the primary endpoint [11,12,13]. In addition, critical assessment revealed that none of the included studies held high scientific quality. 

A basic problem in fatigue research is the lack of a common accepted definition [27]. Indeed, lack of definition and conceptual clarification was also observed in this review, where only two studies provided a definition of fatigue [11,13]. On the other hand, the fatigue definitions presented in those two papers both lacked a clear reference to existing literature. 

Fatigue is frequently reported by patients as well as observed by clinicians in celiac disease [23,25,26]. However, a vast majority of the published literature base their reports merely on clinical consultation rather than rigorous methodological research (i.e., using validated measurement tools). Thus, the actual prevalence of fatigue in celiac disease remains unclear. However, there are indications that the level of fatigue is higher in these patients than in control groups and the background population [10,14]

We were only able to identify one single study that reported on potential socio-demographic and clinical factors associated with fatigue symptoms in celiac disease [11]. Of the factors studied, none were significantly associated with fatigue. However, since data is not shown, it is unclear whether this finding was true for all of the different fatigue measures used in the study. Furthermore, two of the included studies found that fatigue was associated with reduced HRQoL and increased sleep problems, respectively [12,14]. Although there is currently very limited documentation on these associations, this appears to coincide with findings in other patient populations [28,29,30,31,32]. In addition, our review only found one study that was designed as an intervention with fatigue as the primary endpoint. Even though Ciacci et al. [13] conclude that l-Carnitine therapy is safe and effective in ameliorating fatigue in celiac disease, these results should be interpreted with extreme caution. Firstly, the study is hampered by the fact that it does not reach its own power estimates due to a large number of patients not completing the study (21.6%). Secondly, the study does not use fatigue measurement tools that has been adequately tested for validity and reliability. In addition, the absolute difference in mean fatigue VAS between the groups at end of study revealed a small effect size according to Cohen’s d [16,33]. 

Several studies in other populations have shown that anaemia is associated with increased fatigue symptoms [7,34]. The pathogenesis of anaemia-related fatigue remains unclear, but some suggest that abnormalities in energy metabolism play a role in inducing fatigue [35]. Moreover, while some studies have shown that haemoglobin response is associated with meaningful improvements in fatigue, others have not been able to reveal any significant association between the use of erythropoiesis-stimulating agents and fatigue symptom [36,37]. In the current review we were unable to identify studies that specifically looked at anaemia as predictor of fatigue in celiac disease. However, Siniscalchi et al. [11] noted that the included celiac patients in their study had significantly lower haemoglobin levels. A similar observation was reported in Jordá et al. [12]. In the latter study, a significant correlation between worse fatigue scores and lower haemoglobin levels was reported. However, even though Jordá et al. [12] report that their regression analysis show that haemoglobin levels may be involved in the perception of fatigue, the data presented in the paper does not justify such a conclusion. In fact, the dependent variable used in their study was not fatigue, but HRQoL (EQ-5D-VAS). Consequently, the current observation on the potential association between fatigue and anaemia was based on a univariate analysis only.

This review is not without limitations. The fact that we chose to limit our focus to adults and English publications only may have influenced our identification of relevant publications. On the other hand, a strength is the rigorous literature search in several databases, as well as the blinded quality assessment of each of the papers by two of the authors. 

## 5. Conclusions

Although frequently reported in clinical practice, fatigue has been scarcely studied in celiac disease. In addition, existing literature is characterized by significant methodological weaknesses. Consequently, there is an unmet need to understand contributing factors for fatigue as well as the impact of fatigue in celiac disease. 

## Figures and Tables

**Table 1:** Search terms used for literature search.

Fatigue	Coeliac Disease
Fatigue (MeSH)	Coeliac disease
Mental fatigue	
Chronic fatigue	
Tiredness	
Exhaustion	
Weariness	
Vitality	
Asthenia	
Low energy	

**Table 2:** Summary of the included articles.

Study (Ref. No.)	Study Population and Setting	Study Design and Participants	Questionnaires Measuring Fatigue	Strengths and Limitations
Häuser et al., 2006 [10]	A subgroup of members (1000/18,355) from the German Coeliac Society (GZG) ≥18 years was invited to participate Every 18th person on the membership list was invited in order to ensure a geographically representative sample Normative data were collected from the handbooks of the SF-36, GBB-24 and HADS-D Exclusion criteria: <18 years of age	Cross-sectional design Available for analyses: *n* = 446	SF-36 (Vitality subscale) GBB-24 (Fatigue subscale)	L: Fatigue not specified as aim, merely reported as parts of the questionnaire results L: No definition of fatigue L: Sample consisting of members of a patient society only L: Low response rate S: Normative data for comparison L: Self-reported information on comorbidity L: Single centre study S: Validated instruments used L: Coeliac disease diagnosis self-reported only
Siniscalchi et al., 2005 [11]	Caucation adults ≥18 years of age from Campania, Italy, were consecutively recruited from an outpatient clinic Participants divided into two groups (Group 1: Patients on gluten containing diet, Group 2: Patients on gluten-free diet) Control group consisted of volunteers recruited from medical an non-medical hospital staff Exclusion criteria: <18 years of age, lack of informed consent, major psychiatric disease, active thyroid gland disease	Cross-sectional design Coeliac disease: *n* = 130 Control group: *n* = 80	CFS FSS VAS	S: Definition of fatigue. L: Inadequate language L: Groups not comparable due to differences in BMI, Ferritin, Haemoglobin L: No evidence of appropriate matching of groups L: Inadequate statistical control for differences between groups L: Methods used to collect socio-demographic and clinical data is lacking L: Unclear presentation of results L: Procedure for questionnaire handling insufficiently described L: Lack of information about validity and reliability on the study questionnaires, both in Italian and in the target group L: No clear identification and control of potential confounding factors L: Data not presented in line with study aims
Jordá et al., 2010 [12]	Patients with celiac disease seen between March 2008 and April 2009 were prospectively invited to participate in the study Included patients stratified in two groups (Group 1: Following gluten-free diet, Group 2: Untreated)	Cross-sectional study *n* = 51 Group 1: *n* = 38 Group 2: *n* = 13	D-FIS	L: No definition of fatigue. S: Validated fatigue questionnaire used L: Small sample and small subgroups L: Lack of information on recruitment procedure L: Lack of information concerning the collection of socio-demographic and clinical data L: No information on ethical approval L: No information about response rate or number of patients approached for inclusion L: Characteristics differ between groups
Ciacci et al., 2007 [13]	Patients with CD screened for inclusion at the Department of Clinical and Experimental Medicine, Federico II University—Naples, Italy6 0 patients randomized following a 30-day gluten-free diet	Randomized, double blind, parallel study *n* = 60 (l-Carnitine group *n* = 30, placebo *n* = 30)	Scott-Huskisson VAS VSA SF-36 (Vitality subscale)	S: Definition of fatigue S: Randomized groups S: Allocation concealment S: Clear definition of coeliac disease L: Large number of participants did not complete study (*n* = 13 (22%)) L: Single centre L: Lack of calculation of effect size L: No information on validity/reliability of fatigue questionnaires
Zingone et al., 2010 [14]	Adult coeliac disease patients consecutively recruited from September 2009 to March 2010 from Frederico II University (Naples, Italy) Participants divided into two groups (Group 1: Coeliac patients at diagnosis on gluten containing diet. Group 2: Coeliac patients at follow up on gluten-free diet) Gender- and age-matched control group Inclusion criteria: Informed consent, 19–60 years Exclusion criteria: Major psychiatric disease, cancer, pregnancy or children blow 3 years of age	Case Control study Group 1: *n* = 30 Group 2: *n* = 30 Control group: *n* = 30	Fatigue-VAS	L: Fatigue not specified as aim, merely reported as parts of the questionnaire results. L: No definition of fatigue L: No information about response rate or number of patients approached for inclusion L: Large numeric differences in characteristics between coeliac groups L: No control for confounding variables S: Gender- and age-matched controls

Table legends and abbreviations: L; Limitation., S; Strength., SF-36; Short Form-36 Health Survey., D-FIS; Daily Fatigue Impact Scale., FSS; Fatigue Severity Scale., CFS; Chronic Fatigue Syndrome Questionnaire., GBB-24., Geißener Symptom Check List., VSA; Verbal Scale for Asthenia, VSA. CD; Coeliac disease, HADS-D; Hospital anxiety and depression scale – depression.

## References

[1] Ludvigsson J.F., Leffler D.A., Bai J.C., Biagi F., Fasano A., Green P.H., Hadjivassiliou M., Kaukinen K., Kelly C.P., Leonard J.N. (2013). The Oslo definitions for coeliac disease and related terms. Gut.

[2] Ludvigsson J.F., Bai J.C., Biagi F., Card T.R., Ciacci C., Ciclitira P.J., Green P.H.R., Hadjivassiliou M., Holdoway A., van Heel D.A. (2014). Diagnosis and management of adult coeliac disease: Guidelines from the British Society of Gastroenterology. Gut.

[3] Leffler D.A., Green P.H., Fasano A. (2015). Extraintestinal manifestations of coeliac disease. Nat. Rev. Gastroenterol. Hepatol..

[4] Lundin K.E., Wijmenga C. (2015). Coeliac disease and autoimmune disease-genetic overlap and screening. Nat. Rev. Gastroenterol. Hepatol..

[5] Hin H., Bird G., Fisher P., Mahy N., Jewell D. (1999). Coeliac disease in primary care: Case finding study. BMJ.

[6] Sanders D.S., Patel D., Stephenson T.J., Ward A.M., McCloskey E.V., Hadjivassiliou M., Lobo A.J. (2003). A primary care cross-sectional study of undiagnosed adult coeliac disease. Eur. J. Gastroenterol. Hepatol..

[7] Jelsness-Jorgensen L.P., Bernklev T., Henriksen M., Torp R., Moum B.A. (2011). Chronic fatigue is more prevalent in patients with inflammatory bowel disease than in healthy controls. Inflamm. Bowel Dis..

[8] Van Langenberg D.R., Gibson P.R. (2010). Systematic review: Fatigue in inflammatory bowel disease. Aliment. Pharmacol. Ther..

[9] Moher D., Liberati A., Tetzlaff J., Altman D.G. (2009). Preferred reporting items for systematic reviews and meta-analyses: The PRISMA statement. Ann. Intern. Med..

[10] Hauser W., Gold J., Stein J., Caspary W.F., Stallmach A. (2006). Health-related quality of life in adult coeliac disease in Germany: Results of a national survey. Eur. J. Gastroenterol. Hepatol..

[11] Siniscalchi M., Iovino P., Tortora R., Forestiero S., Somma A., Capuano L., Franzese M.D., Sabbatini F., Ciacci C. (2005). Fatigue in adult coeliac disease. Aliment. Pharmacol. Ther..

[12] Jorda F.C., Lopez Vivancos J. (2010). Fatigue as a determinant of health in patients with celiac disease. J. Clin. Gastroenterol..

[13] Ciacci C., Peluso G., Iannoni E., Siniscalchi M., Iovino P., Rispo A., Tortora R., Bucci C., Zingone F., Margarucci S. (2007). l-Carnitine in the treatment of fatigue in adult celiac disease patients: A pilot study. Dig. Liver. Dis..

[14] Zingone F., Siniscalchi M., Capone P., Tortora R., Andreozzi P., Capone E., Ciacci C. (2010). The quality of sleep in patients with coeliac disease. Aliment. Pharmacol. Ther..

[15] Wessely S., Powell R. (1989). Fatigue syndromes: A comparison of chronic “postviral” fatigue with neuromuscular and affective disorders. J. Neurol. Neurosurg. Psychiatr..

[16] Cohen J. (1988). Statistical Power Analysis for The Behavioral Sciences.

[17] Zarkadas M., Cranney A., Case S., Molloy M., Switzer C., Graham I.D., Butzner J.D., Rashid M., Warren R.E., Burrows V. (2006). The impact of a gluten-free diet on adults with coeliac disease: Results of a national survey. J. Hum. Nutr. Diet..

[18] Spijkerman M., Tan I.L., Kolkman J.J., Withoff S., Wijmenga C., Visschedijk M.C., Weersma R.K. (2016). A large variety of clinical features and concomitant disorders in celiac disease—A cohort study in the Netherlands. Dig. Liver Dis..

[19] Silvester J.A., Graff L.A., Rigaux L., Walker J.R., Duerksen D.R. (2016). Symptomatic suspected gluten exposure is common among patients with coeliac disease on a gluten-free diet. Aliment. Pharmacol. Ther..

[20] Jericho H., Sansotta N., Guandalini S. (2017). Extraintestinal manifestations of celiac disease: Effectiveness of the gluten-free diet. J. Pediatr. Gastroenterol. Nutr..

[21] Sansotta N., Amirikian K., Guandalini S., Jericho H. (2018). Celiac disease symptom resolution: Effectiveness of the gluten-free diet. J. Pediatr. Gastroenterol. Nutr..

[22] Catassi C., Kryszak D., Louis-Jacques O., Duerksen D.R., Hill I., Crowe S.E., Brown A.R., Procaccini N.J., Wonderly B.A., Hartley P. (2007). Detection of Celiac disease in primary care: A multicenter case-finding study in North America. Am. J. Gastroenterol..

[23] Nurminen S., Kivela L., Huhtala H., Kaukinen K., Kurppa K. (2018). Extraintestinal manifestations were common in children with coeliac disease and were more prevalent in patients with more severe clinical and histological presentation. Acta Paediatr..

[24] Barratt S.M., Leeds J.S., Sanders D.S. (2013). Factors influencing the type, timing and severity of symptomatic responses to dietary gluten in patients with biopsy-proven coeliac disease. JGLD.

[25] Ford S., Howard R., Oyebode J. (2012). Psychosocial aspects of coeliac disease: A cross-sectional survey of a UK population. Br. J. Health Psychol..

[26] Francavilla R., Cristofori F., Castellaneta S., Polloni C., Albano V., Dellatte S., Indrio F., Cavallo L., Catassi C. (2014). Clinical, serologic, and histologic features of gluten sensitivity in children. J. Pediatr..

[27] DeLuca J. (2005). Fatigue: As a Window to the Brain.

[28] Jelsness-Jorgensen L.P., Bernklev T., Henriksen M., Torp R., Moum B.A. (2011). Chronic fatigue is associated with impaired health-related quality of life in inflammatory bowel disease. Aliment. Pharmacol. Ther..

[29] Frigstad S.O., Hoivik M.L., Jahnsen J., Cvancarova M., Grimstad T., Berset I.P., Huppertz-Hauss G., Hovde Ø., Bernklev T., Moum B. (2018). Fatigue is not associated with vitamin D deficiency in inflammatory bowel disease patients. WJG.

[30] Kotterba S., Neusser T., Norenberg C., Bussfeld P., Glaser T., Dorner M., Schürks M. (2018). Sleep quality, daytime sleepiness, fatigue, and quality of life in patients with multiple sclerosis treated with interferon beta-1b: Results from a prospective observational cohort study. BMC Nephrol..

[31] Rupp I., Boshuizen H.C., Jacobi C.E., Dinant H.J., van den Bos G.A.M. (2004). Impact of fatigue on health-related quality of life in rheumatoid arthritis. Arthritis Rheum..

[32] Opheim R., Fagermoen M.S., Bernklev T., Jelsness-Jorgensen L.P., Moum B. (2014). Fatigue interference with daily living among patients with inflammatory bowel disease. Qual. Life Res..

[33] Cohen J. (1992). A power primer. Psychol. Bull..

[34] Romberg-Camps M.J., Bol Y., Dagnelie P.C., Hesselink-van de Kruijs M.A., Kester A.D., Engels L.G., van Deursen C., Hameeteman W.H.A., Pierik M., Pierik F. (2010). Fatigue and health-related quality of life in inflammatory bowel disease: Results from a population-based study in the Netherlands: The IBD-South Limburg cohort. Inflamm. Bowel Dis..

[35] Sobrero A., Puglisi F., Guglielmi A., Belvedere O., Aprile G., Ramello M., Grossi F.A.O.U. (2001). San Martino —IST, Istituto Nazionale Ricerca sul Cancro (GENOVA) Fatigue: A main component of anemia symptomatology. Semin. Oncol..

[36] Bohlius J., Tonia T., Nuesch E., Juni P., Fey M.F., Egger M., Bernhard J. (2014). Effects of erythropoiesis-stimulating agents on fatigue- and anaemia-related symptoms in cancer patients: Systematic review and meta-analyses of published and unpublished data. Br. J. Cancer.

[37] Cella D., Kallich J., McDermott A., Xu X. (2004). The longitudinal relationship of hemoglobin, fatigue and quality of life in anemic cancer patients: Results from five randomized clinical trials. Ann. Oncol..

